# Improving Kinetic or Thermodynamic Stability of an Azoreductase by Directed Evolution

**DOI:** 10.1371/journal.pone.0087209

**Published:** 2014-01-27

**Authors:** Vânia Brissos, Nádia Gonçalves, Eduardo P. Melo, Lígia O. Martins

**Affiliations:** 1 Instituto de Tecnologia Química e Biológica, Universidade Nova de Lisboa, Oeiras, Portugal; 2 Institute for Biotechnology and Bioengineering, Center for Molecular and Structural Biomedicine, Universidade do Algarve, Faro, Portugal; Jacobs University Bremen, Germany

## Abstract

Protein stability arises from a combination of factors which are often difficult to rationalise. Therefore its improvement is better addressed through directed evolution than by rational design approaches. In this study, five rounds of mutagenesis/recombination followed by high-throughput screening (≈10,000 clones) yielded the hit 1B6 showing a 300-fold higher half life at 50°C than that exhibited by the homodimeric wild type PpAzoR azoreductase from *Pseudomonas putida* MET94. The characterization using fluorescence, calorimetry and light scattering shows that 1B6 has a folded state slightly less stable than the wild type (with lower melting and optimal temperatures) but in contrast is more resistant to irreversible denaturation. The superior kinetic stability of 1B6 variant was therefore related to an increased resistance of the unfolded monomers to aggregation through the introduction of mutations that disturbed hydrophobic patches and increased the surface net charge of the protein. Variants 2A1 and 2A1-Y179H with increased thermodynamic stability (10 to 20°C higher melting temperature than wild type) were also examined showing the distinctive nature of mutations that lead to improved structural robustness: these occur in residues that are mostly involved in strengthening the solvent-exposed loops or the inter-dimer interactions of the folded state.

## Introduction

Thermal stability is relevant for biological function and molecular evolution of proteins. The thermal denaturation process of proteins is usually complex, but often for monomeric proteins can be simplified to the classical two step process: N↔U→D where N, U and D are the native, the reversible unfolded and the irreversible denatured enzyme. The first step involves unfolding of the polypeptide's native structure. The unfolded protein may refold to the native conformation or in a second step undergo irreversible denaturation to permanent inactivation. This may result from protein aggregation, misfolding and covalent changes such as the deamidation of asparagine or glutamine residues and oxidation of cysteine or methionine residues [1]. Enzyme thermostability encompasses thermodynamic and kinetic stabilities [2], [3]. Thermodynamic stability is defined by the enzymes's free energy of stabilization (ΔG_stab_ reflecting the difference between the free energies of the folded and the unfolded states of the protein) and by its melting temperature (T_m_, the temperature at which 50% of the protein is unfolded). Kinetic or long-term stability depends on the energy barrier to irreversible inactivation and is generally expressed as the enzyme's half-life (t_1/2_) at a defined temperature. Most frequently the two stabilities correlate since increasing the enzyme resistance to unfolding (higher T_m_) also increases its resistance to inactivation (higher t_1/2_); firstly, an increase in the stability of the native state leads to slower accumulation of the unfolded state and secondly, the unfolded state is usually the ground state leading to irreversible denaturation/inactivation.

Directed evolution is considered to be the most powerful approach for improving the thermostability of proteins. In fact, comparative studies performed with hyperthermostable enzymes and their mesophilic counterparts have shown nearly superimposable three-dimensional structures suggesting that in nature extreme thermostability seems to be achieved by distributing different types of additional intramolecular interactions throughout the protein [4]. Moreover our understanding of these interactions is incomplete and often does not allow to reliably predicting how they combine to yield a more stable protein. Therefore, rational approaches such as site-directed mutagenesis shows often a limited efficiency and the random introduction of a small number of amino acid changes by error prone PCR or DNA shuffling emerges as the most appropriate methodology to improve protein stability. In recent years these methods have been fine-tuned and a different number of properties in various target enzymes have been successfully improved using directed evolution approaches [5]–[7]. Thermal stability is a critical property for many biotechnological applications of proteins as it implies longer life-times and frequently higher tolerance to the presence of organic co-solvents, extreme pH values and high salt concentration or pressures. Several examples of successfully evolved lipases, β-glucuronidases, ligninolytic oxidoreductases, xylanases, cytochrome P450 peroxygenases, phytases and glucose dehydrogenase have been reported [8]–[15].

Flavin-dependent azoredutases have been identified in a wide range of synthetic dye decolourising bacteria including *Escherichia coli*, *Bacillus* sp. SF, *Bacillus* sp. OY1–2, *Pseudomonas aeruginosa*, *Enterococcus feacalis*, *Salmonella typhimurium* [16]–[20]. Enzymatic bioreduction of synthetic azo dyes or nitroaromatics, prevalent anthropogenic pollutants, are environmental friendly strategies for bioremediation of e.g. dye-containing effluents from textile industries [21]–[23]. These enzymes share some similarities with regard to sequence, structure, and reaction mechanism with the larger family of flavin-dependent quinone reductases such as, Lot6p from *Saccharomyces cerevisiae* or the mammalian NQO1 [24]. Azoreductases are proposed to take part in the organism's enzymatic general detoxification systems; e.g. in the cellular response to thiol-specific stress [25], [26] or in the response to oxidative stress [27], [28]. These enzymes require two cycles of NADPH-dependent reduction of FMN to FMNH_2_ for reducing the azo substrate to two amines and the quinone substrate to a hydroquinone. *Pseudomonas putida* MET94 is a bacteria that degrades a wide range of structurally distinct azo dyes with high efficiency and the azoreductase PpAzoR was shown to play a key role in this process [29]. Its broad substrate specificity makes it attractive for bioremediation processes [30] but its low kinetic stability impairs exploitation of its full potential for environmentally related applications.

In this study, we generated thermostable PpAzoR variants by directed evolution. We investigated the molecular determinants associated with the thermostability of 1B6 variant which reveals a significantly increased kinetic stability when compared to the wild type. Thermoresistant variants (2A1 and 2A1-Y179H) that denature irreversibly similarly to the wild type but show an increased stability of the native state were also characterized. Based in the recently solved crystal structure of the homodimeric wild type enzyme [31] we rationalize the molecular basis behind the increased kinetic or thermodynamic stability of PpAzoR enzyme.

## Materials and Methods

### Bacterial strains, plasmids and media

*Escherichia coli* strain DH5α (Novagen) was used for routine propagation and amplification of plasmid constructs. *E. coli* Tuner (DE3, Novagen) and KRX (Promega) strains were used to express the *ppAzoR* and variant genes cloned in pET-21a (+) plasmid (Novagen). In the Tuner strain the target genes are under the control of T7 promotor, induced by isopropyl β-D-1-thiogalactopyranoside (IPTG) and in the KRX strain the genes are under the control of the rhaP_BAD_ promoter, induced by rhamnose. Luria-Bertani medium (LB) and Terrific Broth medium (TB) were used for the maintenance and growth of *E. coli* strains, supplemented with appropriate antibiotics when needed.

### Growth of recombinant strains and *ppAzor* overexpression

The plasmid pLP-1 [29], containing the *ppAzoR* gene, was transformed into *E. coli* Tuner (DE3, Novagen) or *E. coli* KRX (Promega), producing LOM528 and LOM531 strains, respectively. Single colonies were used to inoculate 20 mL of LB medium supplemented with 100 µg/mL ampicillin, grown overnight at 37°C, 160 rpm. Fresh cultures were transferred to 100 mL of TB medium supplemented with 100 µg/mL ampicillin, in order to start the growth with an OD_600 nm_ = 0.05. Cultures were incubated at 30°C, 160 rpm and when OD_600 nm_≈0.6, 100 µM IPTG was added to LOM528, and 0.1% rhamnose to LOM531. After 24 h of cultivation, cells were collected by centrifugation (10,000 rpm, 15 min at 4°C). The cell pellets were suspended in 1.5 mL of 20 mM Tris-HCl buffer, pH 7.6, containing 5 mM MgCl_2_, 1 U/mL of DNAse I, and 2 µL/mL of a mixture of protease inhibitors: antipain and leupeptin. Cells were disrupted by French Press (Thermo EFC) and then centrifuged at 18,000 rpm for 2 h at 4°C. The supernatants (cell crude extracts) were collected and used to perform enzymatic assays. The protein concentration was determined using the Bradford assay with bovine serum albumin (BSA) as standard. SDS-PAGE electrophoresis was performed to visualize protein overproduction in crude extracts.

### Enzymatic assays

Enzymatic activities were measured using five quinone substrates, anthraquinone-2-sulfonic acid (AQS), 1,4-benzoquinone (BZ), 1,2-dihydroxybenzene (catechol), 2-hydroxy-1,4-naphthoquinone (Lawsone), 1,2-naphthoquinone-4-sulfonate (NSA), at 50–100 µM, in the presence of 250 µM NADPH. Enzymatic assays were performed at 30°C, in 100 mM sodium phosphate buffer, pH 7. The reactions were initiated by the addition of crude extracts and followed by monitoring the decrease in absorbance of NADPH at 340 nm (ε = 6,220 M^−1^ cm^−1^) on a Nicolet Evolution 300 spectrophotometer (Thermo Industries).

### Random mutagenesis by error-prone PCR (ep-PCR) and mutant library construction

Variation in the ppAzoR gene was generated by using ep-PCR. Primers 5′-GGAGAGTCATATGAAACTGTTGC-3′ (PpaF) and 5′-CAACCAAAGGATCCCTTGATCAGG-3′ (PpaR) were used for amplification. Nucleotides for restriction sites of NdeI and BamHI are underlined. ep-PCR was carried out in 50 µL reaction volumes containing 3 ng of DNA template (plasmid pLP-1, where ppAzoR gene [29] and variants are cloned), 0.5 µM of primers, 200 µM of dNTPs, 7 mM MgCl_2_, Taq polymerase buffer, and 5 U of Taq polymerase (Fermentas). The effect of MnCl_2_ was tested at 0.1–0.25 mM concentrations. After an initial denaturation period of 10 min at 94°C, the following steps were repeated for 30 cycles in a thermal cycler (MyCycler™ thermocycler, Biorad): 1 min at 94°C, 1 min at 55°C and 45 s at 72°C followed by a final 10 min period at 72°C. The amplified products were purified using GFX PCR DNA and Gel Band Purification kit (GE Healthcare). The final PCR products were digested with NdeI/BamHI (Fermentas) and cloned into pET-21a (+) (Novagen). Ligations were performed with T4 DNA ligase (Fermentas) using a 1∶8 vector to insert ratio. Reaction mixtures were incubated overnight at room temperature, incubated at 65°C for 10 min, and then used to transform electrocompetent E. coli KRX cells.

### Recombination by DNA Shuffling and mutant library construction

DNA shuffling was performed as described previously with some modifications [32], [33]. Selected genes coding for the ppAzoR variants were amplified by PCR using primers pET21D (5′-CTTCCCCATCGGTGATGTCGGCGATATAG-3′) and pET21R (5′-CCAAGGGGTTATGCTAGTTATTGCTCAG-3′). A mixture containing 200 ng of each parental gene was digested with 2.5 U/mL of DNase I in a 200 mM Tris-HCl buffer, pH 7 with 80 mM MnCl_2_ for 15 min at 15°C in a thermocycler (MyCycler™ Thermal Cycler, Biorad). Digestion was stopped by adding 6 µL of 0.5 M EDTA. The PCR reassembly was carried out in a 20 µL reaction volume containing 3 µL of DNA fragments, 200 µM of dNTPs, NZYProof polymerase buffer and 2.5 U of NZYProof polymerase (NZYTech). After an initial denaturation period of 3 min at 96°C, the following steps were repeated for 45 cycles in a thermal cycler (MyCycler™ thermocycler, Biorad): 1 min at 94°C, 90 s at 59°C, 90 s at 56°C, 90 s at 53°C, 90 s at 50°C, 90 s at 47°C, 90 s at 44°C, 90 s at 41°C, and 1 min + 5 s/cycle at 72°C followed by a final 10 min period at 72°C. The PCR reassembly products were amplified by PCR using primers PpaF (5′-GGAGAGTCATATGAAACTGTTGC-3′) and PpaR (5′-CAACCAAAGGATCCCTTGATCAGG-3′). PCR was carried out in a 50 µL reaction volume containing 1 µL of PCR reassembly products, 1 µM of primers, 200 µM of dNTPs, NZYProof polymerase buffer, and 2.5 U of NZYProof polymerase (NZYTech). After an initial denaturation period of 3 min at 94°C, the following steps were repeated for 20 cycles in a thermal cycler (MyCycler™ thermocycler, Biorad): 30 s at 94°C, 1 min at 55°C, 90 s at 72°C followed by a final 10 min period at 72°C. The amplified products were purified using GFX PCR DNA and Gel Band Purification kit (GE Healthcare). The final PCR products were digested with NdeI/BamHI (Fermentas) and cloned into pET-21a (+) (Novagen) as described above. Ligation reaction mixtures were used to transform electrocompetent E. coli KRX cells.

### Site directed mutagenesis

Single amino acid substitutions were created using the QCM protocol developed by Strategene. Plasmid containing the 2A1 variant gene was used as template and the forward 5′-CCCACGGCCTGGCCCATGGCCCGGAGCAG-3′ and reverse 5′-CCCACGGCCTGGCCCATGGCCCGGAGCAG-3′ primers were used to generate the 2A1+Y179H variant. Plasmid containing the 1B6 variant gene was used as a template and the forward 5′-GGCTGCCGATCCCATCCCCCACTTCTCCG-3′ and reverse 5′-CGGAGAAGTGGGGGATGGGATCGGCAGCC-3′ primers were used to generate the mutant 1B6+A48P. The presence of the desired mutations in the resulting plasmids and the absence of unwanted mutations in other regions of the insert were confirmed by DNA sequence analysis. PCR was carried out in a 50 µL reaction volume containing 3 ng of DNA template, 2 µM of primers, 200 µM of dNTPs, NZYProof polymerase buffer, and 1.25 U of NZYProof polymerase (NZYTech). After an initial denaturation period of 2 min at 95°C, the following steps were repeated for 30 cycles in a thermal cycler (MyCycler™ thermocycler, Biorad): 1 min at 95°C, 1 min at 68°C, 7 min at 72°C followed by a final 10 min period at 72°C. The amplified product was purified using GFX PCR DNA and Gel Band Purification kit (GE Healthcare). The final PCR product was used to transform electrocompetent *E. coli* KRX cells.

### Overexpression of *ppAzoR* variants in *E. coli*

From a fresh agar plate, individual colonies were randomly picked and transferred to a 96 well-plate containing 200 µL of LB medium supplemented with ampicillin (100 µg L^−1^). In order to avoid evaporation, only the interior wells were used for cell growth while perimeter wells were filled with water and the plates were sealed with a foil and a plastic cover, closed with parafilm. Four wells in each plate were used to inoculate the parent strain of each generation. Cultures were incubated at 30°C for 24 h, at 750 rpm in a Titramax 1000 shaker (Heidolph). Twenty microliters of these cultures were used to inoculate new 96 well-plates containing 180 µL TB medium supplemented with ampicillin (100 µg L^−1^) and incubated at 30°C for 3 h at 750 rpm. After this period 0.1% rhamnose was added to induce gene expression and cells were harvested by centrifugation (4,000 rpm for 20 min at 4°C) after 24 h of growth.

### Cell disruption in 96-well plates

The 96 well-plates were submerged in liquid nitrogen and then thawed at room temperature for 5 min. After 3 cycles of freeze and thaw, cell pellets were resuspended in 100 µL of 20 mM Tris-HCl buffer, pH 7.6. After cell disruption, plates were centrifuged at 4,000 rpm for 30 min at 4°C and supernatants (cell crude extracts) used for enzymatic activity measurements.

### High-throughput screening for thermostability

Cell crude extracts (20 µL) were transferred into two replica 96 well-plates. One plate was assayed for initial activity (A_i_) by adding 180 µL of 100 mM sodium phosphate buffer, pH 7 containing 100 µM AQS and 250 µM NADPH. The decrease of NADPH absorption was followed during 5 min at 340 nm (ε = 6,220 M^−1^ cm^−1^) on a Synergy 2 (Biotek) micro plate reader. The crude extracts in the second plate were incubated at different temperatures for a defined time period, cooled on ice for 5 min, incubated at room temperature for 5 min and afterwards assayed for residual activity (A_r_) using the same conditions indicated for assaying the initial activity. Thermostability was assessed using the ratio of the residual activity to the initial activity of the variant (v), normalized to the parent type (p) – (A_r_/A_i_)_v_/(A_r_/A_i_)_p_. The variants activity relative to the parent was calculated using the ratio of the initial activity of the variant to the parent type (Ai_v_/Ai_p_). Variants exhibiting either the highest initial activity or the highest thermostability were re-screened to rule out false positives. Mutations were verified by DNA sequencing analysis using T7 terminator universal primers. In each generation, the variant with the highest stability yet unchanged activity was chosen to be the parent for the next generation and the initial and residual activity was measured in tree different 96-well plates, ensuring the reproducibility.

### Production and purification of selected variants

Plasmids of *ppAzoR* variants were introduced into the host *E. coli* Tuner (DE3, Novagen) and recombinant enzymes were produced and purified as previously described [29]. Enzyme concentration was estimated using the Abs_455 nm_ value [29].

### Kinetic analysis

Enzymatic activities of purified variants were measured at 30°C, in 100 mM sodium phosphate buffer, pH 7, with 100 µM AQS and 250 µM NADPH as substrates. Reactions were initiated by the addition of enzyme and followed by monitoring the decrease in absorbance of NADPH at 340 nm (ε = 6,220 M^−1^ cm^−1^) on a Nicolet Evolution 300 spectrophotometer (Thermo Industries). Optimal temperatures were determined between 23 and 65°C.

### Enzyme stability assays

Residual activities were determined at 30°C after incubating purified enzyme preparations at a range of temperatures (40–85°C) for 1 h. Kinetic stability studies were performed as described by Martins et al. [34]. In brief, the enzymes were incubated at 50°C in 20 mM Tris-HCl buffer, pH 7.6 and, at fixed time intervals, sample aliquots were withdrawn and tested for activity at 30°C. Thermodynamic stability was assessed by steady-state fluorescence measured with a Carry Eclipse spectrofluorimeter using excitation wavelengths of 280 nm and 296 nm and an emission wavelength of 340 nm [35], [36]. Samples containing PpAzoR and its variants (Abs_280_ = 0.1) in 20 mM Tris–HCl buffer, pH 7.6 were placed onto a thermostatically controlled thermal block and then heated at a rate of 1°C/min up to 100°C. For the chemical stability studies, increased guanidinium hydrochloride (GdnHCl) concentrations were used to induce protein unfolding, monitored through a combination of fluorescence intensity and emission maximum as previously described. The thermodynamic stability of both enzymes was analyzed based on a two-state process using the equations described in Durão et al [35] and Fernandes et al [36]. Protein aggregation occurring upon thermal unfolding was monitored by measuring static light scattering at 500 nm as excitation and emission wavelengths. Differential scanning calorimetry (DSC) measurements were carried out by VP-DSC from MicroCal at a scan rate of 1°C/min. The experimental calorimetric trace was obtained with 0.2 mg/mL of protein in 20 mM Tris-HCl buffer, pH 7.6. The data were processed and fitted using Origin® software supplied by the DSC manufacturer. The progress baseline-subtracted and concentration-normalized DSC curve was fitted to non-two-state transitions.

## Results and Discussion

### Validation of high-throughput screenings

The measurement of enzymatic activity in crude extracts of *E. coli* strains overexpressing *ppAzoR* was tested using AQS, BZ, catechol, Lawsone and NSA as substrates. AQS was selected based on the highest activity differences (40 times) in induced *vs.* non induced strains. LOM531 (*E. coli* KRX) strain was selected for further studies since 1) the differences in activity are 8 times higher as compared with LOM528 (*E. coli* Tuner) and 2) KRX is a cloning as well as an expressing strain, allowing avoiding additional steps of plasmid transfer during the laboratory evolution process. Coefficients of variance (CV  =  standard deviation/mean x 100%) of ≈15% were achieved for final OD_600 nm_ of cell cultures as well as for total protein content and maximal rates of enzymatic activity in cell crude extracts using six different 96-well plates, ensuring the aimed reproducibility of the *ppAzoR* expression system. For the set-up of the thermostability assays the temperature and the time of incubation (55°C/60 min for the screening of the 1^st^ generation) were selected based on conditions that resulted in a residual activity of about one-third of the initial activity [37]; the thermostability assays showed a CV of ≈17%.

### Directed evolution of PpAzoR

**Figure 1:**
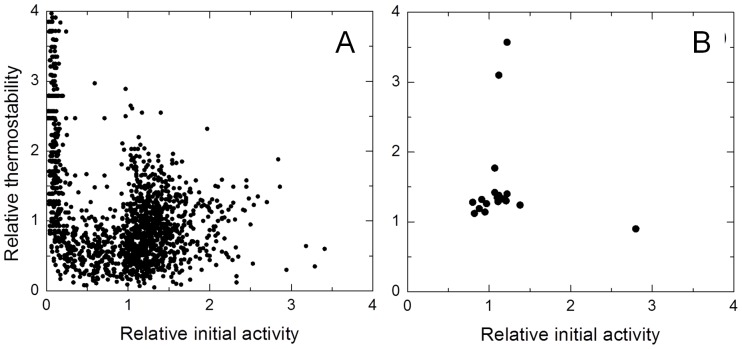
Directed evolution landscape for the first generation mutant library. (A) Initial activity *vs* thermostability of 2214 clones screened relative to the wild type. (B) Re-screening of the best mutants identified. Stability was measured by the ratio of residual activity following incubation at 55°C for 60 min to initial activity.

**Figure 2:**
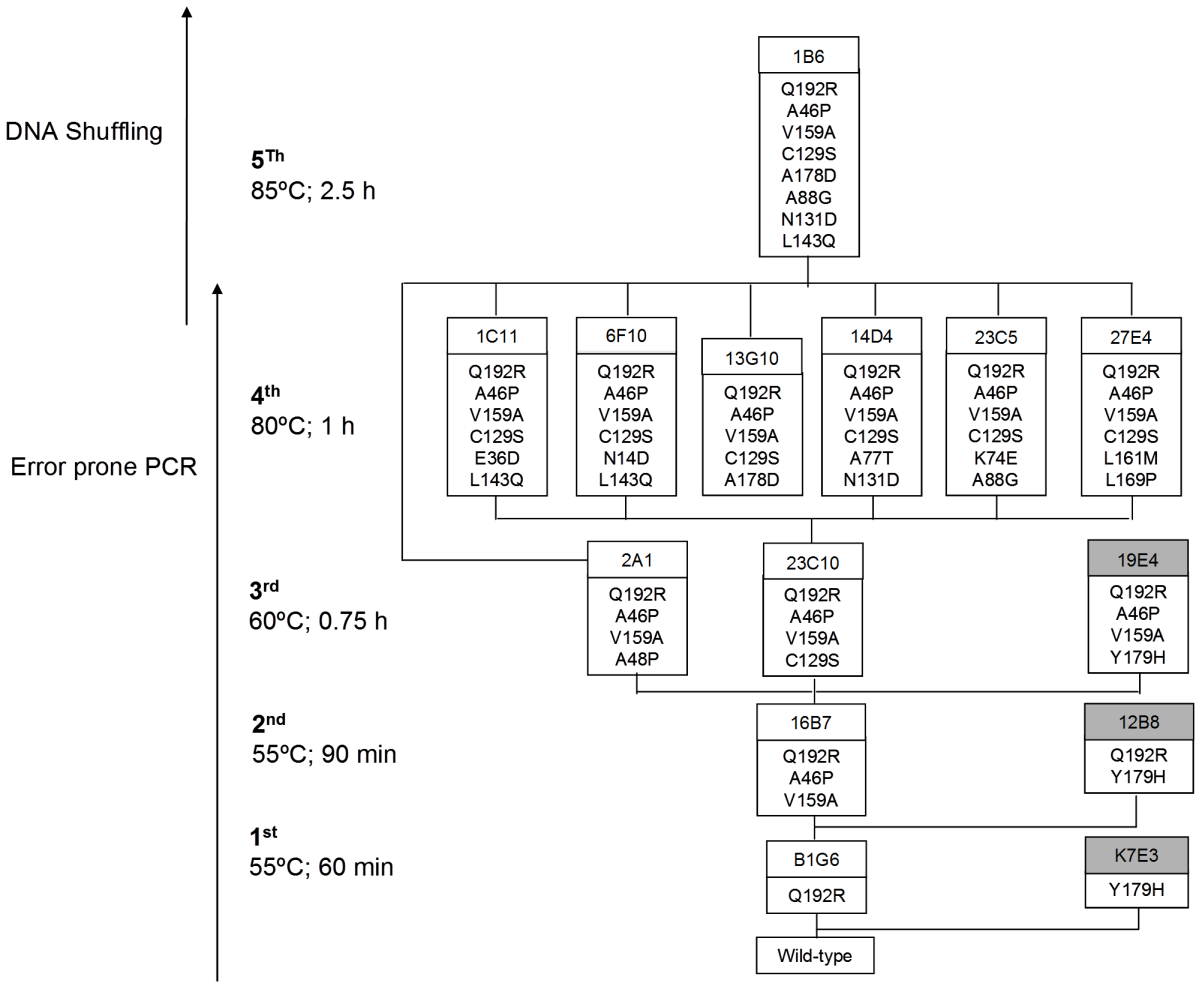
Lineage of PpAzoR variants generated in this study. Only non-synonymous mutations are shown. The mutants with higher stability are in white and the mutants with higher activity are in grey.

**Figure 3:**
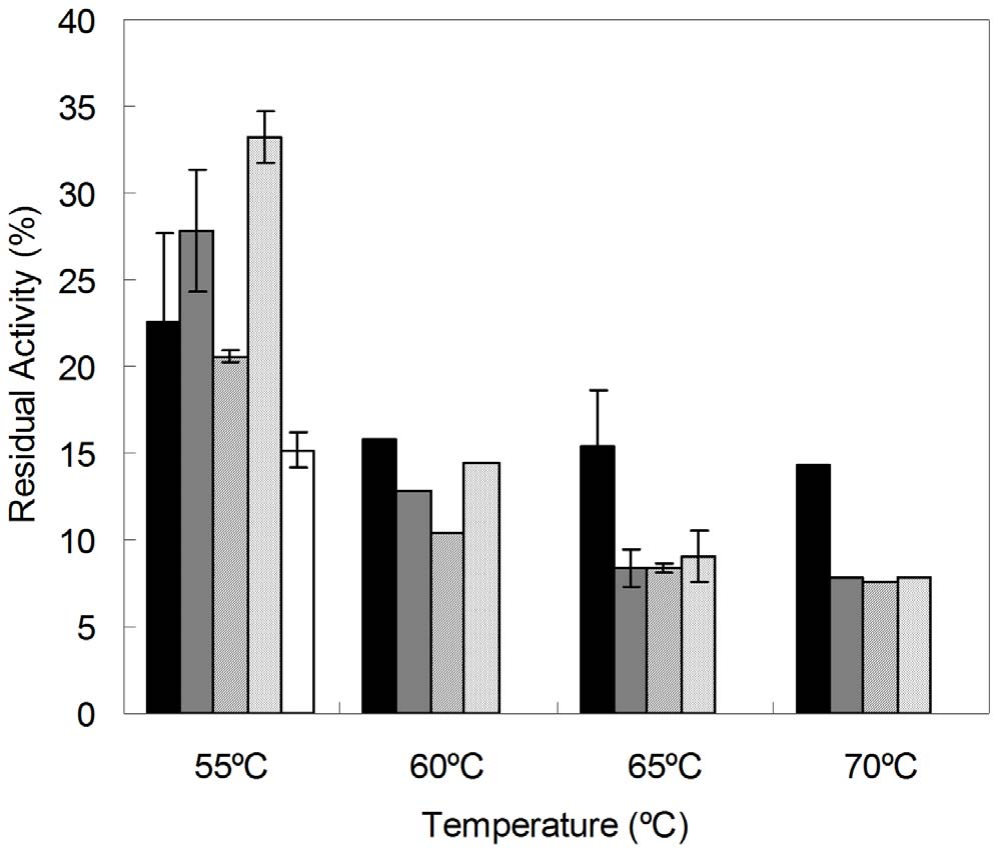
Residual activity of variants from the 5^th^ generation. Activity was measured after incubation at different temperatures (55–70°C) for 30 min: 1B6 (black), 2E4 (grey), 2F11 (diagonal), 6F11 (dot), 3B9 (white). Reactions were performed in 100 mM sodium phosphate buffer pH 7, 100 µM AQS and 250 µM NADPH.

Around 10,000 clones were examined in five rounds of laboratory evolution for improved thermostability. The libraries were constructed to attain 1–3 amino acid changes which correspond roughly to 30 to 45% of the total number of clones with less than 10% activity of wild type [38]. For this purpose MnCl_2_ concentrations were varied and 0.2 mM MnCl_2_ was selected. In the 1^st^ generation a total of 2214 clones were screened (Figure 1A) and 18 variants were identified with increased thermostability or activity and re-screened to rule out false positives (Figure 1B). The B1G6 variant with one amino acid substitution (Q192R) showed around 3.5-fold higher thermostability while maintaining a similar enzymatic activity when compared to the wild type and was selected as parent for the 2^nd^ generation (Table 1 and Figure 2). The heat treatment was adjusted to 55°C for 90 min and 2052 clones were screened and hit 16B7 was identified showing a 3.2-fold higher thermostability as compared with the parent. In the 3^rd^ generation the heat treatment was further adjusted to 60°C for 45 min and the screening of 2160 clones resulted in the identification of two hits, 2A1 and 23C10, exhibiting similar (2.5-fold) improvements in thermostability. The selection of the 23C10 as parent for the 4^th^ generation was based on its higher enzymatic activity in comparison to variant 2A1. Both variants shared mutations Q192R, A46P, V159A from the previous generations and while 23C10 acquired the additional mutation C129S, 2A1 acquired mutation A48P. In the 4^th^ generation the heat treatment was further adjusted to 80°C for 60 min and the screening of 2160 clones resulted in 6 variants with additional 1–3 different new mutations but showing similar thermostability (Table 1). Therefore a recombination approach through DNA Shuffling using the 6 variants and 2A1 from the previous generation was followed (Figure 2). A total of 702 clones were screened with a heat treatment adjusted to 85°C for 150 min and 1B6 was selected after re-screening as the most thermostable variant (Table 1, Figure 3) and was purified.

**Table 1:** Summary of library screening conditions, amino acid substitutions accumulated in PpAzoR variants and initial activity and thermostability relative to their parents.

Generation	Temperature and incubation period	Variants	Mutations	Initial activity relative to parent1)	Thermostability relative to parent2)
1^st^	55°C, 60 min	**B1G6**	Q192R	1.2±0.1	**3.7**±**0.5**
		K7E3	Y179H	**2.8**±**0.2**	0.8±0.3
2^nd^	55°C, 90 min	**16B7**	Q192R, A46P, V159A	0.7±0.1	**3.2**±**0.9**
		12B8	Q192R, Y179H	**5.4**±**0.5**	0.6±0.1
3^rd^	60°C, 45 min	**23C10**	Q192R, A46P, V159A, C129S	1.4±0.1	**2.5**±**0.1**
		2A1	Q192R, A46P, V159A, A48P	0.5±0.2	**2.6**±**0.4**
		19E4	Q192R, A46P, V159A, Y179H	**3.2**±**0.2**	1.4±0.1
4^th^	80°C, 60 min	**13G10**	Q192R, A46P, V159A, C129S, D7H, A178D	0.8±0.1	**3.9**±**0.2**
		**6F10**	Q192R, A46P, V159A, C129S, N14D, L143Q	1.0±0.1	**3.8**±**0.2**
		**27E4**	Q192R, A46P, V159A, C129S, L161M, L169P	0.9±0.1	**3.1**±**0.2**
		**23C5**	Q192R, A46P, V159A, C129S, K74E, A88G	0.9±0.1	**2.9**±**0.4**
		**1C11**	Q192R, A46P, V159A, C129S, E36D, L143Q	1.6±0.4	**2.8**±**0.3**
		**14D4**	Q192R, A46P, V159A, C129S, A77T, N131D	1.0±0.1	**2.5**±**0.2**
		32F5	Q192R, A46P, V159A, C129S, I6V, T79R, Y179H	**2.5**±**0.2**	1.4±0.2
		23E4	Q192R, A46P, V159A, C129S, Y179H	**2.1**±**0.2**	0.9±0.2
5^th^	85°C, 150 min	1B6	Q192R, A46P, V159A, C129S, A178D, A88G, N131D, L143Q	1.8±0.1	**2.2**±**0.2**
		2E4	Q192R, A46P, V159A, C129S, A178D, K74E, L143Q	1.7± 0.01	**2.2**±**0.2**
		2F11	Q192R, A46P, V159A, C129S, A178D, A31S, K74E, A88G, L143Q	1.9±0.1	**2.2**±**0.4**
		6F11	Q192R, A46P, V159A, C129S, A178D, N131D, L143Q	1.7±0.1	**2.3**±**0.2**
		3B9	Q192R, A46P, V159A, C129S, A178D, A77T, F98L, N131D	1.4±0.4	**2.3**±**0.3**

1) Ratio of the initial activity of the variant to the parent type (Ai_v_/Ai_p_),

2) Ratio of the residual activity to the initial activity of the variant (v), normalized to the parent type (p) – (A_r_/A_i_)_v_/(A_r_/A_i_)_p_

The parents for the next generations are in bold.

### Characterization of 1B6, a variant resistant to aggregation

**Figure 4:**
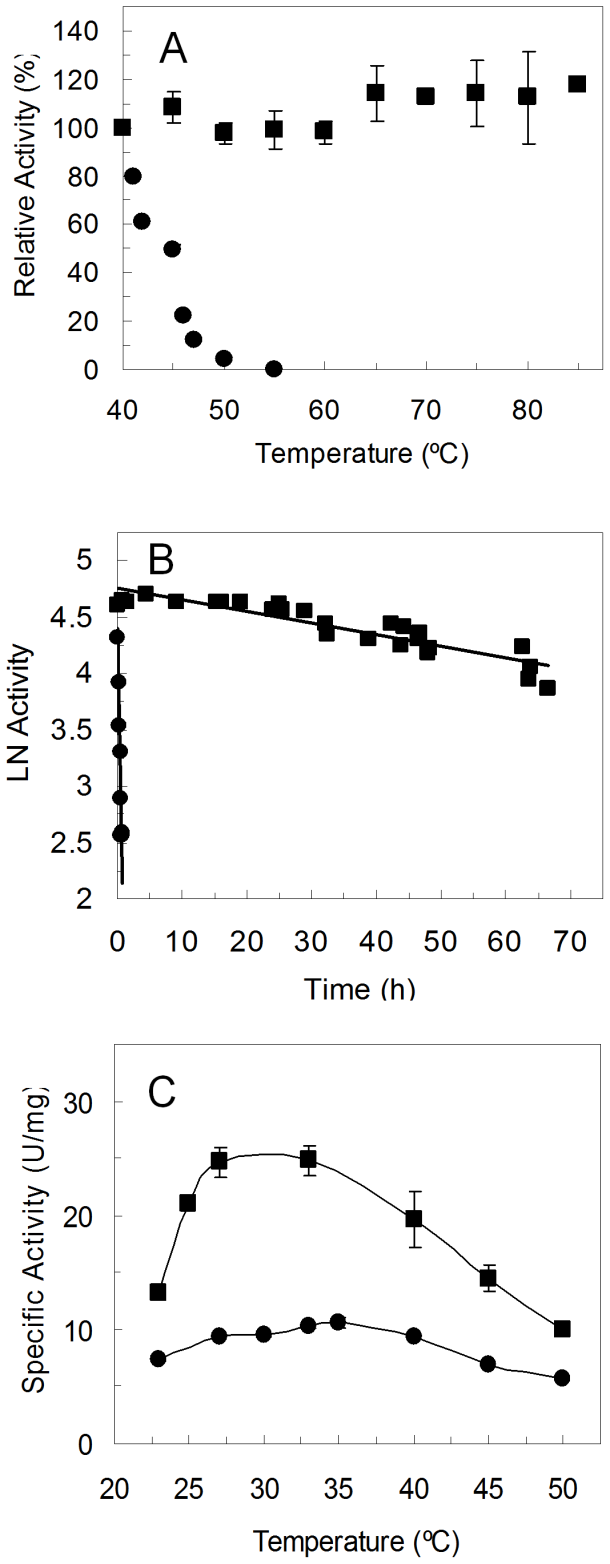
1B6 variant characterization. (A) Activity remaining at 30°C following heating to the indicated temperature for 1 h of wild type PpAzoR (circles) and 1B6 variant (squares). Reactions were performed in 100 mM sodium phosphate buffer pH 7, 100 µM AQS and 250 µM NADPH. (B) Thermal inactivation of wild type PpAzoR (circles) and 1B6 variant (squares). Enzyme samples were incubated at 50°C and activity measured at 30°C in aliquots taken at different time intervals. (C) Temperature dependence of wild type PpAzoR (circles) and 1B6 variant (squares).

Purified 1B6 variant maintains its full activity after incubation for 1 h at temperatures between 40 and 85°C in clear contrast with the wild type that loses activity after one hour at 50–55°C (Figure 4A). First, kinetic stability, that quantifies the amount of enzyme that loses activity irreversibly during incubation at a certain temperature, was measured (Figure 4B). 1B6 is remarkably more stable than the wild type with a 300-fold longer half-life (t_1/2_), i.e. retaining 50% of activity after 68 h at 50°C, while the wild type enzyme half-life is around 13 min. The optimum temperature of 1B6 (around 30°C) is however slightly lower than that of the wild type (around 35°C) (Figure 4C).

**Figure 5:**
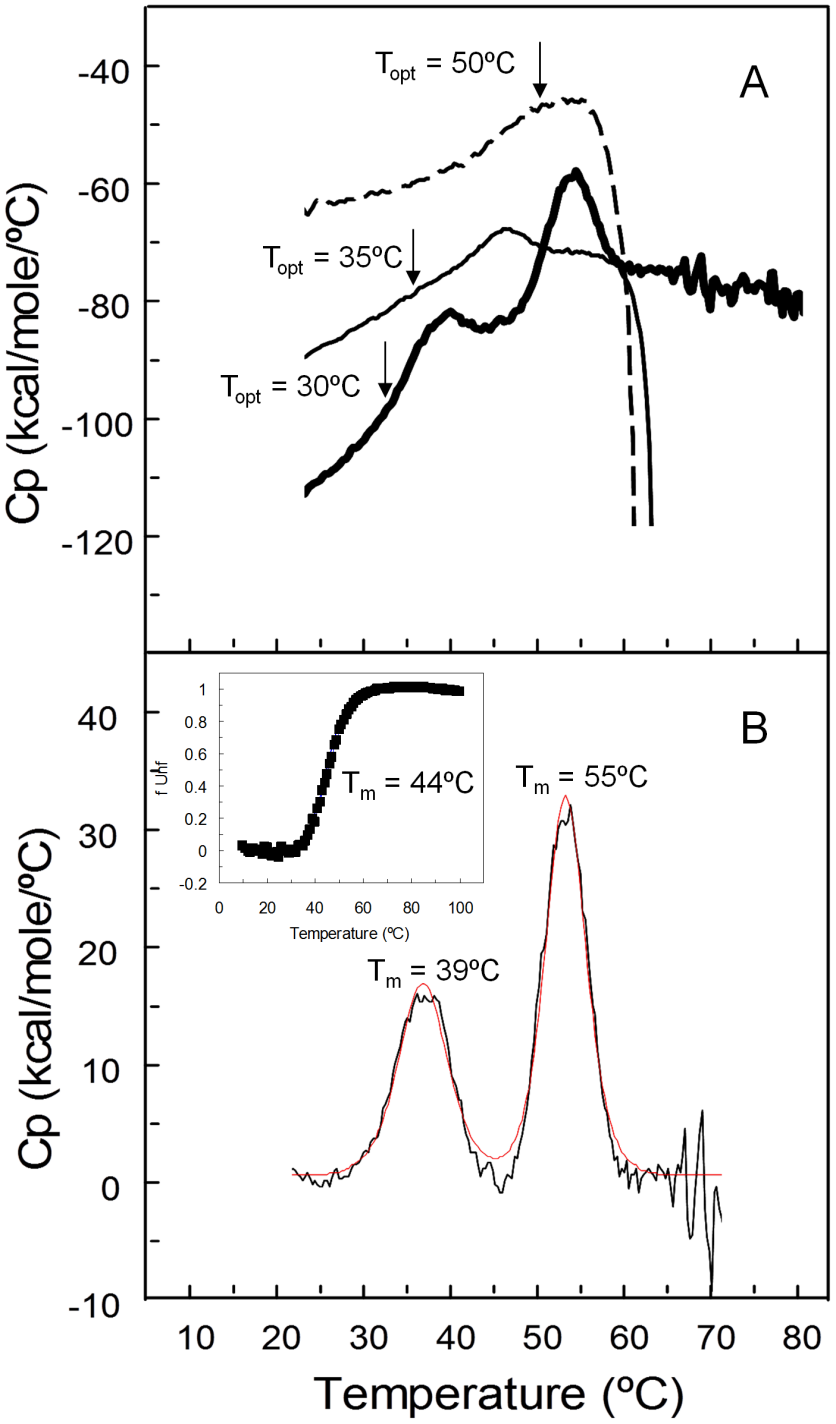
Differential scanning thermograms. (A) Wild type PpAzoR (thin line), 2A1 variant (dash line) and 1B6 variant (thick line). Arrows indicate the optimal temperature for activity which occurs in the initial part of the endothermic peak. (B) 1B6 variant fitted by non-two-state transitions (red line); Inset - Fraction of 1B6 variant unfolded (f_Unf_) by temperature at pH 7.6 as measured by fluorescence emission. The solid line is the fit according to the equation f_U_ = e^(−ΔG°/RT)/(1+e (−ΔG°**/**RT))^.

The thermodynamic stability of wild type enzyme and 1B6 variant was first addressed by differential scanning calorimetry (DSC) (Figure 5A). Besides the measurement of protein unfolding temperatures DSC gives unique information on thermal stability of proteins based on heat changes. The DSC thermogram of wild type shows the typical protein aggregation fingerprint characterized by an abrupt drop of the baseline after the endothermic peak leading to 100% irreversibility, a T_m_∼47°C could be roughly estimated. This feature remains at least up to the 3^rd^ generation of evolution as clearly shown by the profile DSC trace of 2A1 variant (Fig. 5 dashed line). The variant 1B6 does not aggregate after thermal unfolding (no drop in the base line) and displays two distinct endothermic transitions (Figure 5B). For a homodimeric protein as PpAzoR two distinct endothermic transitions were assigned to the dimer dissociation (T_m_(1B6) = 39°C, around 8°C lower than that of wild type) and monomer unfolding (T_m_(1B6) = 55°C). Interestingly, thermal unfolding of 1B6 variant followed by fluorescence, apparently displayed a single transition with a melting temperature of 44±1°C (Figure 5B inset). This T_m_ value is in between dimer dissociation and monomer unfolding temperatures probed by DSC, indicating that tryptophan fluorescence was affected upon dimer dissociation. Moreover, this variant showed nearly complete reversibility of thermal unfolding. The wild type could not be followed by fluorescence due to protein aggregation upon temperature increase. However, two endothermic transitions are still perceptible in the wild type thermogram (Figure 5A). Nevertheless, protein aggregation occurring concomitantly with monomer unfolding abolished almost completely the second transition. The inactivity of the monomer was confirmed by enzymatic assays after its generation by size-exclusion chromatography in the presence of 0.35 M GdnHCl (data not shown).

Based on the data discussed above, thermal deactivation of PpAzoR can be described by the following pathway N_2_↔2N↔2U→D (where N_2_ is the homodimer, N the folded monomer, U the unfolded monomer) that lead to the irreversible aggregate state D in the wild type enzyme. The stabilization of 1B6 variant, leading to the large increase in the kinetic stability, is achieved by preventing the aggregation of the unfolded monomers (i.e. N_2_↔2N↔2U can be proposed for the studied T interval)). A slight simultaneous decrease of the stability of the dimeric folded state of the enzyme (lower optimal temperature (T_opt_) and lower T_m_ of the first endothermic peak) as compared with the wild type was also observed.

### Identification and characterization of thermodynamically resistant variants

**Figure 6:**
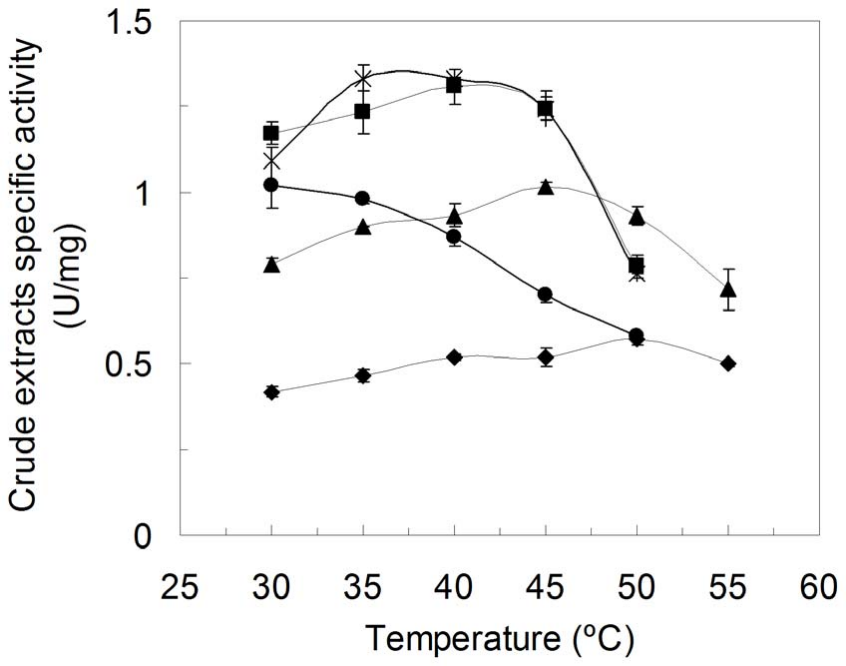
Temperature dependence of PpAzoR wild type and variants measured in crude cell extracts. Wild type PpAzoR (circles), B1G6 from 1^st^ generation (squares), 16B7 from 2^nd^ generation (triangles), 2A1 from 3^rd^ generation (diamonds) and 23C10 from 3^rd^ generation (stars). Reactions were performed using crude extracts in 100 mM sodium phosphate buffer, pH 7, in the presence of 100 µM AQS and 250 µM NADPH.

In order to find hit variants with increased thermodynamic stability and explore further the stability pathways of PpAzoR, we have measured the optimal temperature of several variants found in the 1^st^, 2^nd^ and 3^rd^ generation (Figure 6). An upward shift in the optimum temperature by approx. 10°C in the hit of the 1^st^ generation (T_opt_ B1G6 = 40°C), 15°C in the hit of the 2^nd^ generation (T_opt_ 16B7 = 45°C) and 20°C in one of the hits of the 3^rd^ generation (T_opt_ 2A1 = 50°C) was observed. It is now clear that the similar thermal stabilities measured for 23C10 and 2A1 (Table 2) arise apparently from different phenomena: in 23C10 (T_opt_ of 35–40°C) from the impeded aggregation of the unfolded state and in 2A1 from the stabilization of the folded state.

**Table 2:** Thermodynamic stability of the tertiary structure of PpAzoR wild type, 2A1, 2A1-Y179H and 1B6 variants as assessed by fluorescence spectroscopy.

	Wt	2A1	2A1-Y179H	1B6
**ΔG^water^ (kcal/mol)**	4.2±0.4	7.5±0.7	3.9±0.3	2.3±0.2
***m*** ** (kcal/mol M)**	3.3±0.2	6.9±0.6	3.9±0.3	4.2±0.4
**[GdnHCl]_50%_ (M)**	1.1±0.2	1.3±0.2	1.0±0.3	0.6±0.1

**Figure 7:**
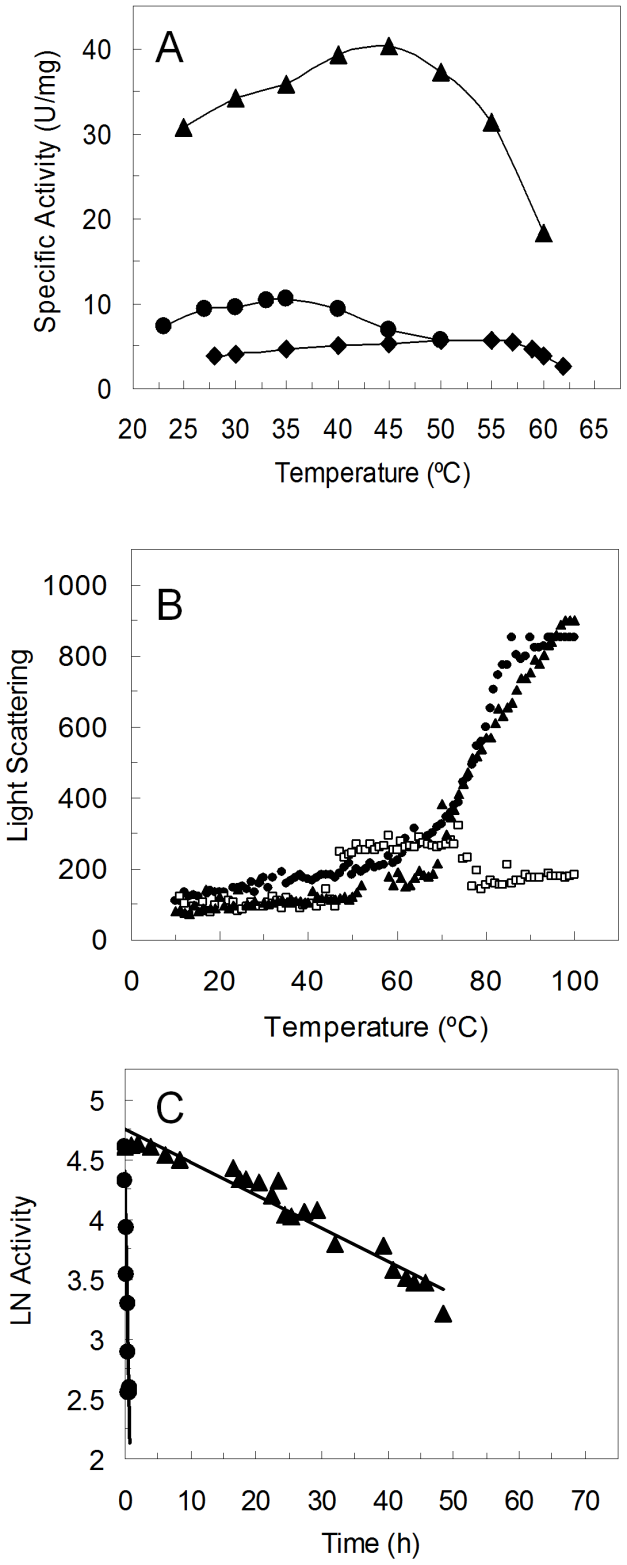
2A1 and 2A1-Y179H variants characterization. (A) Temperature dependence of purified wild type PpAzoR (circles), 2A1 (diamonds) and 2A1-Y179H (triangles) variants. Reactions were performed in 100 mM sodium phosphate buffer, pH 7, in the presence of 100 µM AQS and 250 µM NADPH. (B) Light scattering of wild type PpAzoR (circles), 1B6 (squares) and 2A1-Y179H (triangles) variants. (C) Thermal inactivation of wild type PpAzoR (circles) and 2A1-Y179H (triangles) variant; enzyme samples were incubated at 50°C and activity measured at 30°C in aliquots taken at different time intervals.

The successive improvements in “thermostability” as indicated by the increased optimal temperature of hits in the first generations arises with a simultaneous decrease in activity (Figure 6), revealing a trade-off between high activity and high stability [39]. This is particularly evident in 2A1 variant which, in spite, of the significant up-shift of 20°C of its T_opt_ shows a 2-fold decrease in the enzymatic activity when compared to the wild type enzyme. In order to improve its activity the mutation Y179H was introduced by site-directed mutagenesis since all variants showing increased activity share this mutation (Figure 2 and Table 1). Tyr179 is located in the surroundings of the substrate binding site close to the FMN cofactor and it is likely that the smaller volume of the histidine residue as compared to tyrosine may account for a slight enlargement of the cavity, contributing to an increased activity. Remarkably, the resulting variant 2A1-Y179H indeed shows an 8-fold higher activity than 2A1 corresponding to a 4-fold higher specific activity in relation to wild type (Figure 7A). A compromise between high activity and high stability is also present in 2A1-Y179H since it has a lower optimal temperature in relation to variant 2A1; however it still shows a 10°C up-shift in the T_opt_ in relation to the wild type and 1B6 variant (Figure 7A). Aggregation impeded probing the unfolding process by fluorescence in 2A1 and 2A1-Y179H variants but static light scattering confirmed that unlike 1B6 variant, 2A1 variant as wild type aggregate at the highest temperatures tested (Figure 7B). Furthermore, we observed only one endothermic peak in the DSC thermogram, suggesting the simultaneous occurrence of dimer dissociation and monomer unfolding with a increased melting temperature of around 8°C over that of the wild type (T_m_∼55°C, Figure 5A). Overall, our data reveal that although variant 2A1-Y179H unfolds irreversibly similarly to the wild type, it shows a higher stabilization of the dimeric folded state with a higher melting temperature (and thus up-shifted T_opt_) and 100-fold increased half-life at 50°C (t_1/2_ = 23 h; Figure 7C).

### Characterization of chemical stability of variants

**Figure 8:**
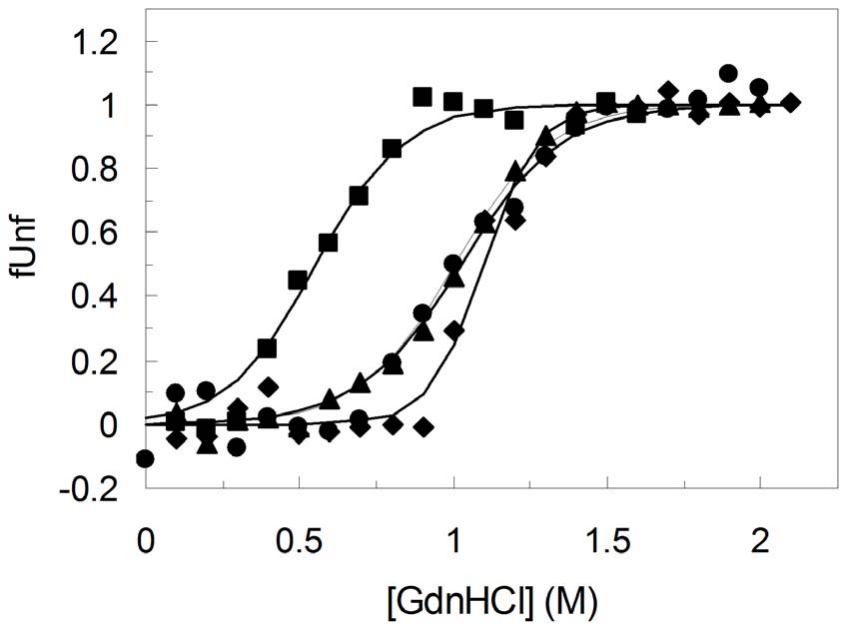
Chemical stability. Unfolded fraction (f_Unf_) of wild type PpAzoR (circles) and 1B6 (squares), 2A1 (diamond) and 2A1-Y179H (triangles) variants by GdnHCl at pH 7.6 as measured by fluorescence emission. The solid line is the fit according to the equation f_U_ = e^(−ΔG°/RT)/(1+e (−ΔG°**/**RT))^.

The thermodynamic stability of the tertiary structure of PpAzoR wild type and hit variants upon addition of GdnHCl was assessed by fluorescence (Figure 8). The wavelengths at the emission maxima reflect clearly the exposure of tryptophan residues to the high polarity of water molecules at the surface of the protein upon unfolding [40]. The wild type PpAzoR displays a GdnHCl concentration of 1.1 M at the mid-point (where 50% of the molecules are unfolded) and the native state is more stable than the unfolded state by 4.2 kcal/mol at 25°C (Table 2). The unfolding process can be accurately described according to a two-state process with folded and unfolded states of the monomers being the only ones that accumulate significantly. Hit variants follow approximately the same trend upon increased GdnHCl concentrations, however noteworthy 50% of the 1B6 molecules unfolded at half of the GdnHCl concentrations required to unfold the wild type. The native state of 1B6 is more stable than the unfolded state by only 2.3 kcal/mol. This result shows that mutations leading to the drastic reduction of the irreversible aggregation result in an overall folded structure which is less stable than the wild type enzyme, as also observed previously for thermal inactivation by DSC (Figure 5). Variant 2A1 is clearly more stable against chemically induced unfolding displaying the largest free-energy difference between the folded and the unfolded state (7.5 kcal/mol). This large free-energy gap may result from a more restricted conformational freedom (lower entropy) of the unfolded state due to the introduction of two proline residues (A46P and particularly A48P which is not present in variant 1B6). Interestingly the cooperativeness of the unfolding transition (*m* parameter) also increases very significantly in the variant 2A1. This parameter is a measure of the degree of solvent exposure for a given transition; the higher value in 2A1 shows that the level of the solvent exposure is larger than in the wild type and in the other variants analysed. Since the conformational freedom of the unfolded state may be restricted by the introduction of the two prolines, the larger *m* value may be rationalized by the higher degree of compactness of variant 2A1 in the folded state. These two residues are located in the interface of the two monomers [31] and the introduction of a proline in position 48 most probably enhances the homodimer stabilization. The higher exposition to the solvent (*m* value) observed in the variant 2A1 may then relate to an almost simultaneous dimer dissociation and chemical unfolding while in the other proteins the dissociation of the homodimer occurs first followed by monomer unfolding in accordance with DSC data (Figure 5A). The replacement of Y179 by a histidine residue in this variant, despite the improvement in activity, results in decreased free energy differences and cooperativeness of transition (*m*) to values similar to wild type.

### Molecular details of mutations that improve stability

PpAzoR (PDB code 4C0W) is a homodimer and its tertiary structure adopts a flavodoxin-like fold characterized by a central twisted five parallel β-sheet connected by α-helices, which flank the sheet from the front and the back [31], [41]. The arrangement of the α-helices and β-stands is identical to structures of azoreductases from *E. coli* (PDB code 2Z98) [16] *Pseudomonas aeruginosa* (PDB code 2V9C) [20], *Enterococcus feacalis* (PDB code 2HPV) [17] and *Salmonella typhimurium* (PDB code 1T5B). Moreover, it contains the conserved motif patterns of flavin-dependent azoreductases, i.e. the sequence involved in the binding of FAD/FMN cofactors, the sequence involved in the dimerisation of the two monomers of the enzyme and the possible putative NAD(P)H binding motif [29], [31].

**Figure 9:**
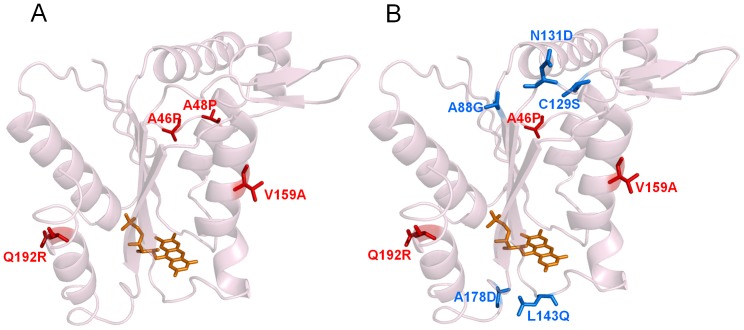
Mapping of key amino acids for protein thermostabilization in PpAzoR crystal structure (PDB code 4C0W). (A) 2A1 and (B) 1B6 variants. Mutations responsible for the thermodynamic stability are in red; mutations responsible for aggregation-resistance are in blue; the FMN is in orange.

All the mutations identified in both the aggregation or thermodynamically-resistant hit variants are spread on or near the surface of the protein (Figure 9), underscoring the importance of protein surface for stability as found in most of the enzymes showing improved thermostability [42], [43]. In addition the majority of mutations occur in loops in accordance with previous studies that show that reduction of flexibility of surface loops generates more stable proteins [44], [45]. It is expected that the less ordered regions, loops and turns, would be more accommodative to the substitutions while disruption of packing in the core may interfere with the folding of the protein.

Aggregation-resistant 1B6 hit shares mutations with thermodynamically-resistant 2A1 variant introduced in the early generations of directed evolution (Figure 9). The substitution Q192R from the 1^st^ generation (Figure 2 and Table 1) most probably relates to the well documented deamidation of glutamine and asparagine residues triggered by high temperatures that contributes to inactivation of irreversibly denaturated proteins [4]. This substitution in an α-helix has an additional advantage as arginine is known to enhance polar interactions, formation of hydrogen bonds and/or salt bridges, resulting in an increased overall stability. A46P and V159A mutations were picked up in the 2^nd^ generation. V159A is located in a α-helix and the replacement of a valine by a smaller alanine, considered to be the best helix-forming residue, may enhance stability by increasing the helical propensity [4]. The A46P mutation occurs in the longest stretch of amino acid residues (20) of the enzyme without any regular secondary structure [31]. In protein structures, loops are flexible regions, and protein unfolding supposedly starts from loops. Proline can adopt only a few conformations showing the lowest conformational entropy among amino acids, restricting therefore the backbone configurations which result in the stabilization of the native state [46]–[48]. The hits from the 1^st^ and 2^nd^ generations have increased optimal temperatures in respect to that of the wild type (Figure 6) therefore it is expectable that they also presented higher melting temperatures. Therefore we can conclude that the mutations Q192R, A46P and V159A stabilised mainly the dimeric native state of the enzyme.

Variant 2A1 from the 3^rd^ generation showing the highest melting temperature and stability of the dimeric native state carries a second Pro (A48P) in the same loop as A46P. A fraction of this loop (Ile47, His49, Phe50) is involved in a complex net of hydrogen bonding interactions with the same stretch of residues of the second monomer of the PpAzoR dimer [31]. Thus, it is probable that Pro48 apart from imposing constraints in backbone conformations of the native state, by decreasing flexibility, contribute critically to the stabilization of the homodimer in solution in accordance with the DSC (Figure 5A) and chemical stability (Figure 8) results.

The aggregation-resistant 1B6, the variant with the longest half-life and dramatically less aggregation-prone, shows not A48P but C129S mutation (Figure 2 and Table 1), located in the putative NAD(P)H binding motif [31]. Cysteines are the most reactive amino acids in proteins which is the reason behind their involvement in specific interactions within proteins (e.g., disulfide bridges or metal ligand binding) and/or their localization in positions inaccessible to the solvent [4]. Importantly, serine with a hydroxyl group allows for the potential formation of additional hydrogen bonds within the protein structure or with the solvent. Interestingly, three out of four mutations introduced in the 4^th^ generation are substitutions to surface charged (N131D, A178D) or polar (L143Q) residues. Notably, five out of the six hit variants from the 4^th^ generation have at least one mutation introducing a charged residue and all variants from the 5^th^ generation have 3 to 4 charged residues in a total of 7 to 8 mutations (Table 1). It has been proposed that the presence of charged residues on the protein surface may lead to residual repulsive interactions increasing the solubility and preventing native state aggregation [49]. The introduction of the positively charged residue R28 was identified as the key determinant of aggregation resistance of the unfolded state of human antibody variable domains created by directed evolution [50] while A281E and V221D mutations inhibited protein aggregation and facilitated refolding of the catalytic active conformation of a *Candida antartica* lipase [51]. In the case of a lipase from *Bacillus subtilis* the simultaneous improvement in thermodynamic stability and non-aggregating properties was achieved by the introduction of two proline residues that led to a better anchoring of loops, disturbing the exposed hydrophobic patches of 6B lipase and one charged residue, E134 [52]. Moreover, we observe here that mutations L143Q and A178D in 1B6 variant are close to a hydrophobic patch present on the protein surface in the surroundings of the FMN molecule, potentially decreasing the association of exposed hydrophobic patches and the formation of aggregate compatible structures. Therefore the combination of the increased surface charge/polarity with disturbance of hydrophobic patches even though resulting in a decreased inter-dimer association, i.e. lower stability of the native state, lead to the abolishment of the aggregational pathway with a critical gain in the kinetic stability of the enzyme. In an attempt to create an aggregation and thermodynamically-resistant variant we have introduced by site-directed mutagenesis the mutation A48P, present in the 2A1, into the 1B6 variant. In contrast to the 1B6, the resulting variant 1B6-A48P aggregates upon temperature raise while maintaining a similar T_opt_ (data not shown). These results suggest the absence of a synergistic interaction between A48P mutation and the mutations present in 1B6 variant, in accordance with our DNA-shuffling results. In fact, none of the five variants selected in the 5^th^ generation as a result of the shuffling among variant 2A1 and variants of the 4^th^ generation, including A48P mutation (Table 1 and Figure 2). Most probably the surface mutations of 1B6, which are not present in 2A1, and A48P comprise an opposite effect in the stability of the native state of the variants since the endothermic transitions in the DSC assigned to dimer dissociation occur at ∼55°C for 2A1 and at 39°C for 1B6 (i.e. 8°C higher and lower, respectively) than the wild type Tm ∼47°C.

## Conclusions

Directed evolution is an effective and reliable approach to engineer proteins contributing simultaneously to our understanding of protein function and adaptation mechanisms. Two types of protein stability (thermodynamic and kinetic) are of interest from a fundamental and applied perspective. Increasing the thermodynamic thermostability is the main issue when an enzyme is used under denaturing conditions (i.e. high temperatures or organic solvents) [2]. Industrial applications require active enzymes rather than enzymes that are in a reversibly inactivated state. For other enzymes, for example those that can be utilized for diagnostic puroposes, to ensure a suitable shelf-life, it is often long term stability that needs to be improved. Most frequently both stabilities correlate since increasing the enzyme resistance to unfolding state also increases its resistance to inactivation. Remarkably in the present study we have identified a hit variant of PpAzoR, 1B6, with increased resistance to inactivation by aggregation. This was accomplished by drastically decreasing the rate of the irreversible step of denaturation (the aggregation of the unfolded monomers) upon thermal stress. Although variant 1B6 is less stable for thermal or chemically induced unfolding when compared to the wild type, lower amounts of the irreversibly denatured state accumulated in the long-term stability assay. The identification of this hit correlates well with the experimental screening strategy followed. In order to select for mutations that improve the stability of the catalytically competent native state a secondary screen strategy was implemented based on activity measurements at increasing temperatures i.e. by probing the dissociation of the dimer. Importantly our work shows that the resistance of the unfolded monomers to aggregation (resulting also in a destabilization of the dimeric folded state) is achieved through a delicate disturbance of hydrophobic patches and to a clearly increased surface net charge. On the other hand, the increase of robustness of the native structure is mainly an outcome of strengthening of solvent-exposed loops and inter-dimer interactions (Table 3). Overall, this work suggests that protein charge can be exploited to impart robust resistance to protein aggregation with implications on de novo protein design efforts, where unpredictable protein properties, including aggregation, remain a significant challenge. From the point of view of protein evolvability mutations that prevent the aggregation of the unfolded state, increasing thus the level of soluble protein, contribute to protein fitness [53], and therefore variant 1B6 is potentially a good candidate to be evolved for new functions of PpAzoR.

**Table 3:** Mechanisms of protein thermostabilization present in PpAzoR variants created by directed evolution [4], [46]–[48], [54]–[57].

Protein thermostabilization	Mutation
**Increasing structure robustness**	
replacing temperature-sensitive residues by polar or charged residues	C129S; Q192R; N131D
strengthening solvent-exposed loops by restricting the number of available main-chain conformations	A46P; A48P
dimer stabilization	A48P
increasing helical propensity	V159A
conformational strain release	A88G
**Resistance to aggregation**	
residual repulsive interactions between partially unfolded structures	N131D; L143Q; A178D
disruption of surface hydrophobic patches	L143Q; A178D
